# A comprehensive drought monitoring method integrating multi-source data

**DOI:** 10.7717/peerj.13560

**Published:** 2022-07-05

**Authors:** Xiaoliang Shi, Hao Ding, Mengyue Wu, Mengqi Shi, Fei Chen, Yi Li, Yuanqi Yang

**Affiliations:** 1 College of Geomatics, Xi’an University of Science and Technology, Xian, Shaanxi, China; 2 No. 6 Geological Party, Guangdong Geological Bureau, Jiangmen, Guangdong, China

**Keywords:** Drought, GRACE, Comprehensive drought index, Climate change, Yellow River Basin

## Abstract

Droughts are the most expensive natural disasters on the planet. As a result of climate change and human activities, the incidence and impact of drought have grown in China. Timely and effective monitoring of drought is crucial for water resource management, drought mitigation, and national food security. In this study, we constructed a comprehensive drought index (YCDI) suitable for the Yellow River Basin using principal component analysis and the entropy weight-AHP method, which integrated a standardized precipitation evapotranspiration index (SPEI), self-calibrating Palmer drought severity index (scPDSI), vegetation condition index (VCI), and standardized water storage index (SWSI). SWSI is calculated by the terrestrial water storage anomaly (TWSA), which can more comprehensively reflect the impact of surface water resources on drought (as compared with soil moisture-based indexes). The study results showed that: (1) compared with single drought index, YCDI has stronger ability to monitor drought process. In terms of time scale and drought degree, the monitoring results based on YCDI were similar with data presented in the China Flood and Drought Bulletin and Meteorological Drought Yearbook, reaching ~87% and ~69%, respectively. The correlation between drought intensity and crop harvest area was 0.56. (2) By the combined analysis of the Mann-Kendall test and Moving T test, it was found that the abrupt change of YCDI index at the time of 2009, mainly due to the precipitation in 2009 reached the lowest value in the past 30 years in northern China and extreme high temperature weather. (3) The YCDI of Henan and Shandong provinces in the middle and lower reaches of the basin decreased more significantly, with the maximum value reaching 0.097/yr, while the index in the upper reaches showed an increasing trend with the maximum rate of 0.096/yr. (4) The frequency of mild drought, moderate drought, severe drought and extreme drought in the Yellow River basin during the study period was 15.84%, 12.52%, 4.03% and 0.97%, respectively. Among them, the highest frequency of droughts occurred in Ningxia, Inner Mongolia and central Shaanxi provinces. Drought causation in the Yellow River basin is more influenced by human activities than climate change in the middle and lower reaches, while climate change is the main factor in the upper reaches. Overall, YCDI is a reliable indicator for monitoring the spatial and temporal evolution of drought in the Yellow River basin, and it can be used for monitoring soil moisture changes and vegetation dynamics, which can provide scientific guidance for regional drought governance.

## Introduction

Climate change and human activities have resulted in the frequent occurrence of hydrological extremes characterized by drought (Huang et al., 2019; Xiao et al., 2016). Drought is a naturally recurring hazard, different from other natural hazards since it often has a slow onset and it is difficult to forecast. The frequency, intensity, and duration of extreme drought events have increased since the early 2000s from global warming and during El Niño periods. As such, the living environment for human beings is compromised and the stability of the economy and society are threatened (Yue et al., 2015). China’s climate is warming and drying, and the frequency and duration of droughts in the north are predicted to increase to all-time highs (Ayantobo, Li & Song, 2019; Dai et al., 2020; Shakeel et al., 2019; Trenberth et al., 2013; Zhu et al., 2018). Thus, timely and accurate monitoring of drought occurrence, severity, and spatial range are of significance.

According to different scientific perspectives, four types of droughts can be identified: meteorological, agricultural, hydrological, and socioeconomic drought (Leng, Tang & Rayburg, 2015). Meteorological drought is defined as the phenomenon of a water shortage caused by the imbalance between evaporation and precipitation, and the water loss is greater than the water gain in a certain period (Zhang et al., 2022). Agricultural drought is defined as the water deficit caused by the shortage of soil moisture during the growth period of crops, which affects the normal growth and development of plants (Zhou et al., 2021). Hydrological drought is caused by the long-term shortage of precipitation, resulting in a reduction of river flows, lake water levels, and reservoir impoundments (Loon & Anne, 2015). Socio-economic drought is defined as an abnormal water shortage caused by the imbalance between the supply and demand of water resources in natural systems and human socio-economic systems (Zhao et al., 2019). The social demand for water can be divided into industrial water demand, agricultural water demand, and water demand for living and service industries. The above four types of droughts are closely intertwined.

A drought index can quantify the complex mechanisms of drought from the perspectives of duration, strength, and severity (Mishra & Singh, 2010). However, due to the differences in drought characteristics around the world, scholars from various countries have developed numerous drought indexes and several drought information systems (*e.g*., measuring severity or magnitude) (Liu et al., 2020; Pulwarty & Sivakumar, 2014). At one time, researchers primarily defined drought as a decrease in precipitation over a certain period (such as with a Z index or the precipitation anomaly percentage) (Rahmati et al., 2020; Yu et al., 2020). The focus now is increasingly on water supply and demand differences, which links water deficit with duration and further supplements the concept of drought (such as Standardized Precipitation Index (SPI) and SPEI) (Erfurt et al., 2020; Yimam et al., 2021). The Palmer Drought Index, proposed in 1965, has been widely used in meteorology, hydrology, water resources, climate, agriculture, and other fields (Palmer, 1965). All drought indexes, however, are constrained by data sources and their regional application is limited by locations of particular weather stations.

The construction and calculation of drought indexes is a critical foundation for studying regional drought quantitatively. The meteorological drought index, the hydrological drought index, the agricultural drought index, and the socio-economic drought index are the most commonly used drought indexes in the international community. They are based on the relative deficit of water in the water cycle, that is, the corresponding form of drought and the classification of drought (Andrew et al., 2022). The drought indicator based on insufficient precipitation is the most common since it is easy to understand, simple, and intuitive. However, it has a long response time, a low sensitivity, and a poor portrayal of drought severity, such as precipitation anomaly percentage, SPI, drought Z index, and so on (Hurtado et al., 2021; Wu et al., 2001). Although a measure based on surface water deficits such as runoff distance level percentage and natural water area distance level percentage can more accurately depict the actual drought conditions, it lacks real-time and comprehensive observation data (Jeng et al., 2005). The index based on soil water deficit include soil relative humidity and soil effective water storage. These indexes can better reflect agricultural drought conditions and related drought losses, but the raw data is difficult to obtain and the accuracy is low. The percentages of groundwater level spacing based on groundwater shortages can better reflect actual drought situations, although observation data is lacking. The standardized precipitation evapotranspiration index, the integrated meteorological drought index (He et al., 2015), the integrated hydrological drought index (Yang et al., 2015a), and the Palmer drought index based on overall water deficit are constructed from several factors in the hydrological cycle, which can explain the drought phenomenon and level from the perspective of water conversion process (Palmer, 1965). In general, none of the current drought indexes have strong physical meaning. Therefore, developing comprehensive drought indexes is an important challenge for drought monitoring.

The establishment of a comprehensive drought index is of great importance for drought monitoring. In recent years, many researchers have attempted to create a comprehensive drought index that integrates multiple drought-related hydrometeorological variables. Considering the needs of different agencies for drought monitoring, integrated drought indexes are often preferred (Huang et al., 2015; Huang et al., 2019; Zhang et al., 2019). Comprehensive drought indexes take into account meteorological, hydrological, and agricultural drought factors. The methods used to combine drought indexes include weight combination, multivariate joint distribution, and machine learning (Andrew et al., 2022; Han et al., 2021a). The raw data for establishing integrated drought indexes is also broadly divided into two categories: one using basic data such as meteorology, vegetation, and soil, and the other using existing drought indexes (Shrestha et al., 2020; Wang et al., 2021).

By referring to other research results, we found that SPEI, scPDSI, VCI, and SWSI all have their own advantages for different types of drought monitoring. The SPEI integrates precipitation and evapotranspiration, allowing it to reflect not just the distribution and trend of regional wet and dry, but also variations in drought at different scales, which can reflect various sorts of drought conditions (Yang et al., 2017). The scPDSI calculation takes into account the entire water balancing process, including precipitation, soil water content, runoff, and potential evapotranspiration, and can more correctly describe drought conditions in a given region (Zhang et al., 2022). The VCI can better depict water stress status and not only monitor and track regional drought but also explain spatial and temporal changes in vegetation, rendering it the optimum data for large-scale remote sensing drought monitoring, and its reliability has been proved by a large amount of data (Zhang et al., 2017). The SWSI is calculated based on information on changes in surface water, soil moisture and groundwater storage, and thus is of great use in monitoring and assessing global and regional droughts (Tapley et al., 2019). As a result, we selected four typical drought indexes to create a comprehensive drought index for the Yellow River basin in this research. A combined weight scheme of subjective and objective weights was used, based on entropy weight-Analytic Hierarchy Process (AHP), to determine reliable weight values of multiple indexes.

Understanding the temporal and spatial variation of drought in the Yellow River Basin in the context of climate change is critical for water resource management and agricultural production in inland China (Piao et al., 2010). The goal of this study was to introduce a general drought definition. This general definition can improve drought monitoring, help decision-makers better allocate water resources, and reduce the impact of drought on different economic and social sectors. The specific objectives of this study are to: (1) Construct the YCDI of the Yellow River Basin using the entropy weight-AHP weight method, based on SPEI, scPDSI, VCI and SWSI; (2) validate the comprehensive drought index (YCDI) of the Yellow River Basin using typical drought events with the China Flood and Drought Bulletin and Meteorological Drought Yearbook; (3) use trend analysis, mutation analysis, and drought frequency analysis to investigate the temporal and spatial variation of drought in the Yellow River Basin; (4) explain the causes of drought in the Yellow River Basin and assess the impact of human activities and climate change on drought disasters.

## Materials and Methods

### Study area

**Figure 1:**
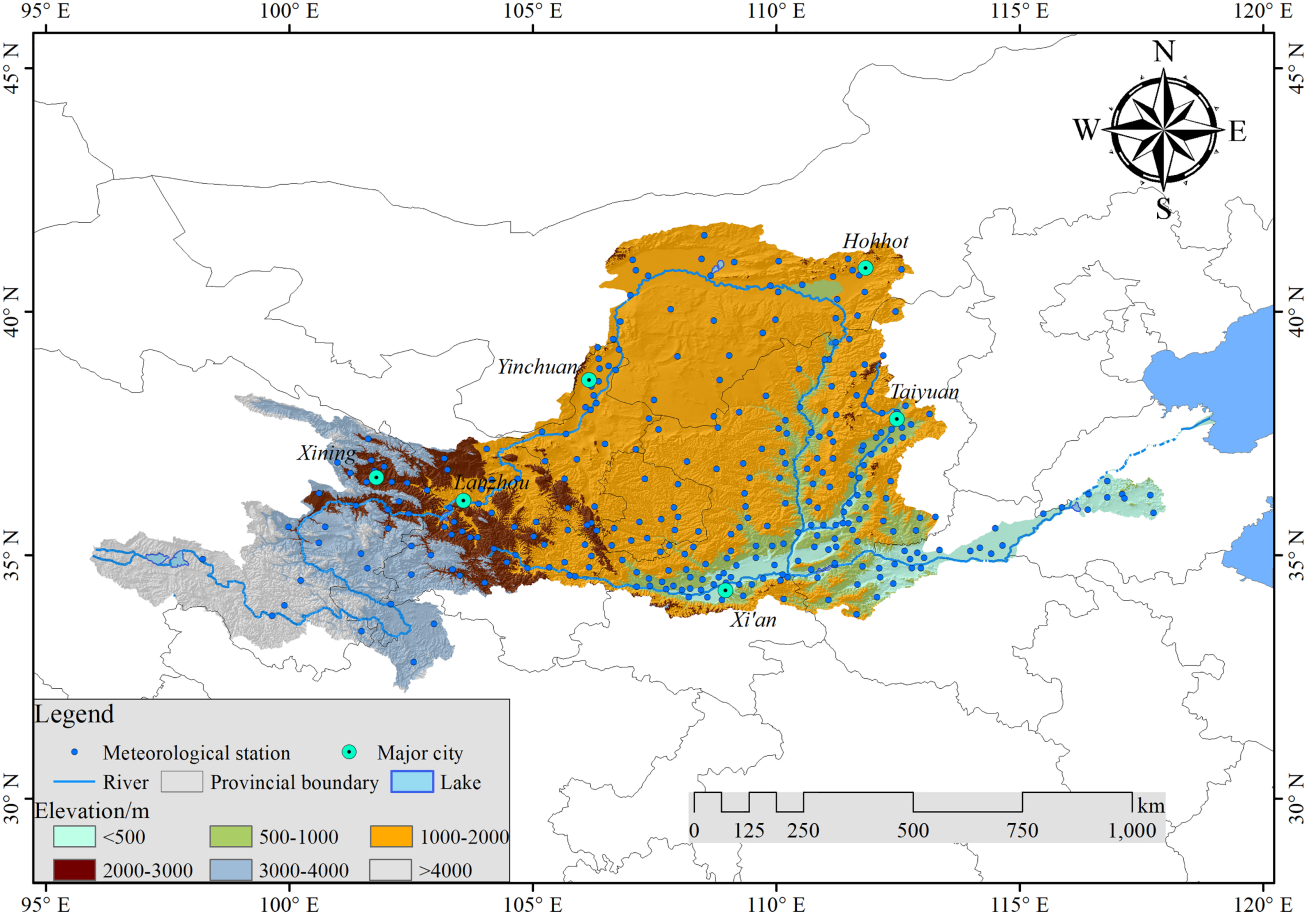
Location of the Yellow River Basin and distribution of meteorological stations.

The Yellow River Basin is located 32–42°N and 96–119°E, a drainage area of 795,000 km^2^, as shown in Fig. 1 (Lu et al., 2021). The Yellow River is the second-longest river in China with a length of 5,464 km. It originates from the Tibetan Plateau, discharges into the Bohai Sea, and flows through nine provinces (Qinghai, Sichuan, Gansu, Ningxia, Inner Mongolia, Shaanxi, Shanxi, Henan, and Shandong) (Aoa et al., 2020). There are many mountains in the Yellow River Basin, and the elevation difference between east and west is large. From west to east are found the Qinghai-Tibet Plateau, the Loess Plateau, and the Huang-Huai-Hai Plain. It is rainy in summer and autumn, the annual precipitation is 123–1,021 mm, and the average annual precipitation is about 378 mm (Huang, Wang & Chen, 2022). The precipitation from June to September accounts for about 70% of the year, and the annual evaporation is 700–1,800 mm. There are four climatic regions: arid, semi-arid, semi-humid, and humid. The Yellow River irrigates 15% of the cultivated land, supports 12% of the country’s population, and drives 14% of the country’s gross domestic product (Wang et al., 2012). As such, it is known as the Mother River of China. However, in recent years, the challenges regarding balancing the supply and demand of water resources in the Yellow River Basin have become prominent, and drought problems have become serious.

### Data

Remote Sensing data: The MODIS NDVI data (MOD13A3) with 1 km spatial resolution was downloaded from https://ladsweb.modaps.eosdis.nasa.gov for 2002 to 2015.

Drought event data: The historical drought events from 2006 to 2015 were obtained from the China Flood and Drought Bulletin published by the Ministry of Water Resources of the People’s Republic of China (http://www.mwr.gov.cn/sj/tjgb/zgshzhgb). The Flood and Drought Bulletin records the occurrence and development of drought, as well as intensity, scope, and associated disasters.

Meteorological Data: The monthly total precipitation data and monthly average temperature data for 2002–2015 were provided by the Meteorological Data Sharing Service System of China Meteorological Administration (http://data.cma.cn/).

scPDSI dataset: The scPDSI dataset (self-calibrating Palmer drought severity index based on Penman-Monteith) came from the Climate Research Unit (CRU) of the University of East Anglia (http://www.cru.uea.ac.uk/data); the spatial resolution is 0.5° × 0.5° and the period from 1901 to 2017.

GRACE data: This paper uses Gravity Recovery and Climate Experiment (GRACE) Level-2 gravity field model data provided by the Center for Space Research (CSR) of the University of Texas (https://www.csr.utexas.edu). Removed from the data are the effects of solid tides, sea tides, polar tides, non-tidal atmosphere and ocean mass generated by the Earth’s rotation, and the gravity disturbance caused by extraterrestrial stars. Finally, details of the datasets used in this study for drought indexes were given in Table 1.

**Table 1:** Datasets use in this study.

Data	Spatial resolution	Temporal resolution	Link
scPDSI	0. 5°	Monthly	http://www.cru.uea.ac.uk/data
Drought event data		Year	http://www.mwr.gov.cn/sj/tjgb/zgshzhgb
Meteorological data		Monthly	http://data.cma.cn/
NDVI	1 km	Monthly	https://ladsweb.modaps.eosdis.nasa.gov
GRACE	0. 5°	Monthly	https://www.csr.utexas.edu

## Methods

### TWSA changes from GRACE

GRACE estimates of mass variations over land reflect primarily related to changes in TWS (including snow/ice, soil moisture, and groundwater), plus other unmodeled signals, such as those related to postglacial rebound (PGR) or tectonics (Han et al., 2019; Jyolsna, Kambhammettu & Gorugantula, 2021). CSR GRACE data are regularized, spherical, harmonic coefficients that exclude the effects of solid tides, sea tides, solid polar tides, marine polar tides, non-tidal atmospheres and seas, and gravity disturbances generated by planets like the sun and moon (Yang et al., 2015b). The SLR coefficient replaces the C_20_ term and the first-order term is rectified. The 60-order coefficient is reduced in this article to prevent the impact of high-order noise and odd-even-order correlation error. Gaussian filtering and the P3M15 reverse boundary extension were employed for processing. The mass variations were then converted to TWSA in units of equivalent water height (EWH) (Scanlon et al., 2016). The WGS 1984 datum were used to project the 1° × 1° grid data.

(1) $$\eqalign{TWSA & = {{a{\rho _{ave}}} \over {3{\rho _{water}}}}\sum\limits_{l = 0}^\infty {\sum\limits_{m = 0}^l {{P_{lm}}} } \left( {\cos \theta } \right){{2l + 1} \over {1 + {k_l}}} \cr & \cdot \left( {\Delta {C_{lm}}\cos \left( {m\lambda } \right) + \Delta {S_{lm}}\sin \left( {m\lambda } \right)} \right)lm{P_{lm}}\left( {\cos \theta } \right){k_l}}$$

In the formula, $TWSA$ is the monthly water reserve change, $a$ is the equatorial radius; ${\rho _{ave}}$ is the mean density of the Earth; $l$, $m$ are the order and number of the Earth’s gravity field, ${P_{lm}}\left( {\cos \theta } \right)$ is the fully specified connective Lejeune function; ${k_l}$ is the load Lejeune number corresponding to order $l$; $\Delta {C_{lm}}$, $\Delta {S_{lm}}$ are the normalized spherical harmonic coefficient variables; $\theta$ is the calculation point residual is the longitude of the calculation point; $\lambda$ is the longitude of the calculation point.

### The single drought index calculation

SWSI is a hydrological drought index, which is used to evaluate the loss state of water storage change (Araghinejad, 2011). Its formula is:

(2) $${\rm{SWSI}} = {{{\rm{TWS}}{{\rm{A}}_{{\rm{i}},{\rm{j}}}} - {\rm{TWS}}{{\rm{A}}_{{\rm{j}},{\rm{mean}}}}} \over {{\rm{TWS}}{{\rm{A}}_{{\rm{j}},{\rm{std}}}}}}$$

In the formula, $TWS{A_{i,j}}$ is the monthly water reserve change, $TWS{A_{j,mean}}$ is the monthly water reserve change mean, and $TWS{A_{j,std}}$ is the monthly water reserve change standard deviation.

For vegetation regions, the weather-related Normalized Difference Vegetation Index (NDVI) changes are smaller than the ecosystem-related ones and the drought impacts on vegetation cannot be easily detected from NDVI data directly (Quiring & Ganesh, 2010). Thus, Kogan (1995) developed a vegetation condition index to control local differences in ecosystem productivity, which is used to reflect the state of vegetation in different growth periods. It is defined as:

(3) $$VCI = {{NDV{I_i} - NDV{I_{\min }}} \over {NDV{I_{\max }} - NDV{I_{\min }}}}$$

where *NDVI*_*i*_ is the monthly normalized vegetation index, *NDVI*_*max*_ and *NDVI*_*min*_ are monthly normalized vegetation index maximums and minimums over the study period. VCI ranges from 0 to 1 corresponding to the changes in vegetation condition from unfavorable to optimal.

The SPEI is a regional meteorological drought monitoring index proposed by Vicente-Serrano (Vicente-Serrano, Beguería & López-Moreno, 2010). The index represents regional drought conditions by calculating the deviation degree of the difference between precipitation and potential evapotranspiration from the average state (Beguería et al., 2014). Considering the influence of precipitation and temperature on drought, the calculation is:

(4) $$SPEI = \left\{ \matrix{
  w - {{{c_0}\; - \;{c_1} \times w\; + \;{c_2}\; \times \;{w^2}} \over {1\; + \;{d_1}\; \times \;w\; + \;{d_2}\; \times \;{w^2}\; + \;{d_3}\; \times \;{w^3}}} \hfill \cr 
  w = \sqrt { - 2\ln \left( p \right)} ,p \le 0.5 \hfill \cr 
   - \left( {w - {{{c_0}{\mkern 1mu} {\mkern 1mu}  - {\mkern 1mu} {\mkern 1mu} {c_1}{\mkern 1mu} {\mkern 1mu}  \times {\mkern 1mu} {\mkern 1mu} w{\mkern 1mu} {\mkern 1mu}  + {\mkern 1mu} {\mkern 1mu} {c_2}{\mkern 1mu} {\mkern 1mu}  \times {\mkern 1mu} {\mkern 1mu} {w^2}} \over {1{\mkern 1mu} {\mkern 1mu}  + {\mkern 1mu} {\mkern 1mu} {d_1}{\mkern 1mu} {\mkern 1mu}  \times {\mkern 1mu} {\mkern 1mu} w{\mkern 1mu} {\mkern 1mu}  + {\mkern 1mu} {\mkern 1mu} {d_2}{\mkern 1mu} {\mkern 1mu}  \times {\mkern 1mu} {\mkern 1mu} {w^2}{\mkern 1mu} {\mkern 1mu}  + {\mkern 1mu} {\mkern 1mu} {d_3}{\mkern 1mu} {\mkern 1mu}  \times {\mkern 1mu} {\mkern 1mu} {w^3}}}} \right) \hfill \cr 
  w = \sqrt { - 2\ln \left( {1 - p} \right)} ,p{\mkern 1mu} {\mkern 1mu}  > {\mkern 1mu} {\mkern 1mu} 0.5 \hfill \cr}  \right.$$

where the constant terms *c*_*0*_, *c*_*1*_, *c*_*2*_, *d*_*1*_, *d*_*2*_, and *d*_*3*_ are 2.515517, 0.802853, 0.010328, 1.432788, 0.189269, and 0.001308; *w* is the cumulative probability-weighted moment; the cumulative probability *P* is the normalized result of the different data series of precipitation and potential evapotranspiration, where the calculation of potential evapotranspiration adopts the Penman formula.

### Weight of the drought index calculation

The corresponding grid values were extracted from the meteorological stations, and 11,952 groups of grid values from April 2002 to December 2015 were used as weight samples. The weights of four indexes were calculated based on the principle of entropy weight-AHP weight method. Finally, the weights of each index in the comprehensive index were calculated by combining the two weights (Ni et al., 2009; Xie, Gu & Lin, 2014). The following calculation is used:

(5) $${\omega _i} = {\lambda\omega{^\prime_i}} + \left( {1 - \lambda } \right){\omega^{\prime \prime}_i}$$

where ${\omega _i}$ is the comprehensive weight of the first evaluation index and $\lambda$ is the preference coefficient ( $\lambda$ = 0.5).

### Aggregation

To create a monthly comprehensive drought index in the Yellow River Basin, a single drought index is integrated using the weight calculated by the formula (4). The equation is:

(6) $$YCDI = {\omega _1}SPEI + {\omega _2}scPDSI + {\omega _3}VCI + {\omega _4}SWSI$$

where ${\omega _i}$ is the weight corresponding to a single drought index.

### Drought frequency calculation

According to the classification of YCDI (Table 2), the frequencies of meteorological drought days for each level were calculated by the following equation:

**Table 2:** Weight of each drought index.

	SPEI	scPDSI	VCI	SWSI
*ω’*_*i*_	0.251	0.243	0.231	0.275
*ω’’*_*i*_	0.224	0.249	0.239	0.288
*ω*_*i*_	0.237	0.245	0.237	0.281

(7) $${P_i} = {{{n_i}} \over N}$$

where *N* is the total months (165 months), *n*_*i*_ is the total number of different droughts, and *i* is the drought level (extreme drought, severe drought, moderate drought, mild drought) (Felicia, Omid & Amir, 2021).

### Run theory

The primary priority of run-length theory is to uncover the statistical law of continuous occurrence of random events and calculate the probability distribution and return period of continuous durations quantitatively (Moyé & Kapadia, 1995). It is a useful theoretical method for figuring out the basic law of drought occurrence. Based on the YCDI calculation results, three characteristic variables of drought factors were separated using the run theory method from the YCDI sequence: drought frequency, drought duration, and drought intensity. During the drought process, drought intensity (s) refers to the accumulation of the difference between the drought index value and the drought threshold, and drought duration (t) refers to the time from the start to the end of the drought. Drought duration and intensity are two terms that can be used to describe and reflect the severity of a drought (Tayyebeh et al., 2020; Wang et al., 2020).

### Mann-kendall and moving T test

The Mann-Kendall nonparametric statistical test is recommended by the International Meteorological Organization (WMO) for time series trend analysis of environmental data and is a tool for testing the trend of drought time series. The Mann-Kendall nonparametric test was used to analyze the significance of YCDI changes in the Yellow River basin, and when |Z| > 1.96, the significance test with confidence level α = 0.05 was passed. For the specific calculation of Mann-Kendall can be referred to Yue & Wang (2004). A Moving T test was also introduced in this study to improve the accuracy of the mutation test and eliminate the erroneous mutation sites created by the M-K test. For the specific calculation of Moving T test can be referred to Joses et al. (2019).

## Results

### Verification of the comprehensive drought index in the yellow river basin

Based on the calculation of the principal component analysis-entropy weight method, the weights of drought indexes are shown in Table 2.

According to the weight of each drought index, the comprehensive drought index model of the Yellow River Basin is:

(8) $$YCDI = 0.2705\;SPEI + 0.245\;scPDSI + 0.2155\;VCI + 0.269\;SWSI$$

Based on the above formula and the observation data of 79 meteorological stations in the Yellow River Basin, we selected 13,035 YCDI values as our samples. The SPEI and other comprehensive drought index (Tayyebeh et al., 2020; Vicente-Serrano, Beguería & López-Moreno, 2010; Yu et al., 2020) were classified according to the national standard of Meteorological Drought Grades, combined with relevant research results and the frequency of drought occurrence in different grades in the Yellow River Basin. According to the requirements of 2% special drought, 5% severe drought, 10% medium drought, 15% light drought, and 68% no drought, five grades are shown in Table 3.

**Table 3:** Drought classification.

Drought grades	Near normal	Mild drought	Moderate drought	Severe drought	Extreme drought
YCDI	>0	(−0.7,0]	(−1.1, −0.7]	(−1.4, −1.1]	−1.4<

**Figure 2:**
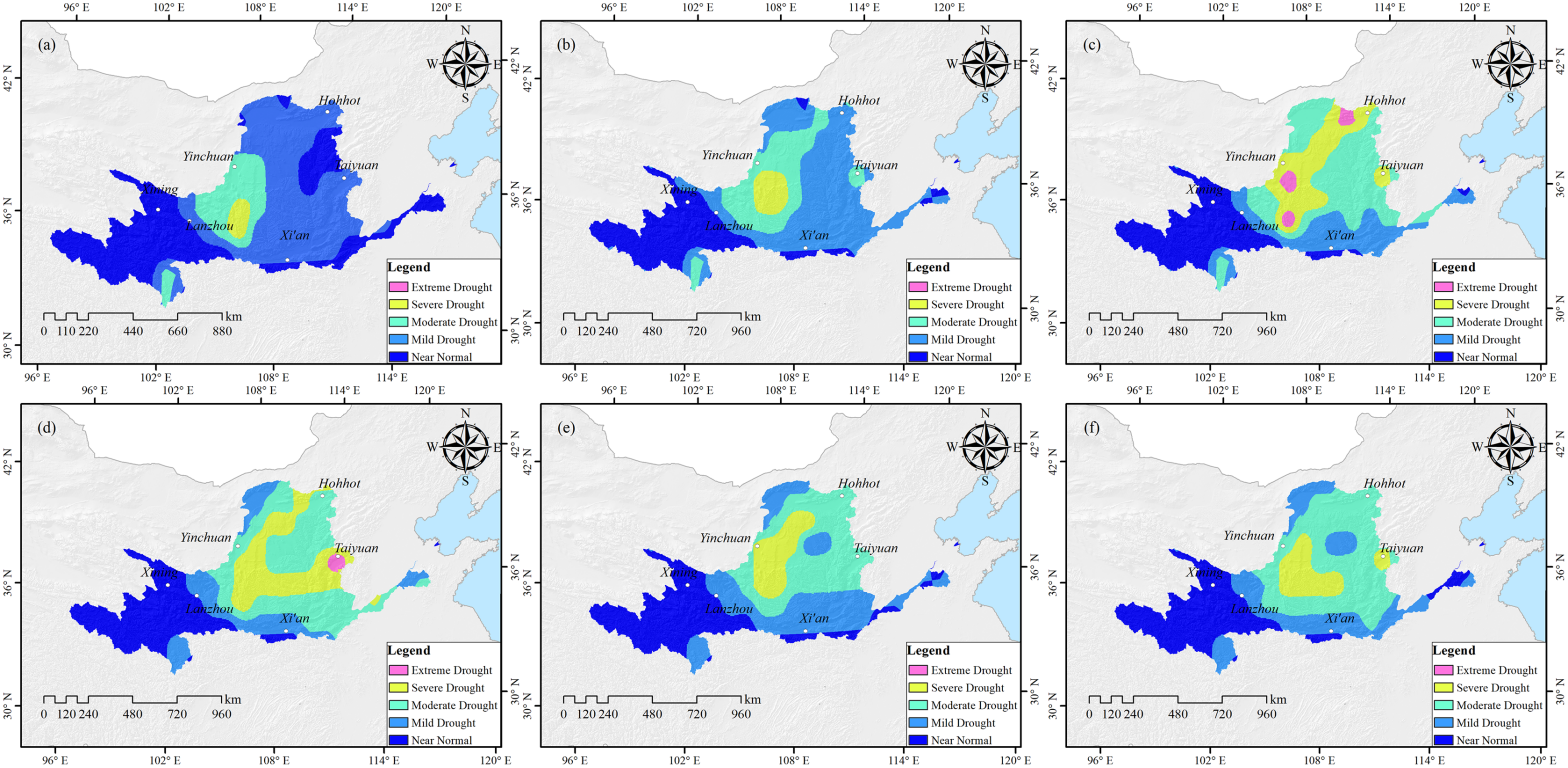
Drought monitoring in the Yellow River Basin from October 2008 to March 2009 based on the YCDI. (A) October 2008. (B) November 2008. (C) December 2008. (D) January 2009. (E) February 2009. (F) March 2009.

According to the 2009 China Flood and Drought Bulletin, a drought occurred in China’s winter wheat region in December 2008 and lasted until February 2009, with severe drought areas persisting until March. From October 2008 to March 2009, the YCDI monitored the drought development. The drought began in the Ningmeng reach, eastern Gansu, and western Shaanxi in October 2008. The drought spread to the southeast in November and December, with the range reaching its peak in January 2009. The drought was alleviated from February to March 2009 because of increased precipitation in the basin Fig. 2.

The China Flood and Drought Bulletin and Meteorological Drought Yearbook were used as standards to verify the monitoring accuracy of YCDI. The results showed that the accuracy of YCDI in drought monitoring could reach ~87%. (Table 4). In addition, we also verified the drought level and drought intensity. First, we compared the drought levels monitored by YCDI with the officially reported drought levels, and the agreement between them reached ~69% (Table 4). Second, we used the area of crop extinction in major provinces in the Yellow River basin from 2006 to 2015 as a metric of drought intensity to verify the accuracy of the YCDI drought intensity. Table 5 shows the coefficients of correlation between crop extinction area and drought intensity for each province in the Yellow River Basin (Note: Drought did not occur in Shandong and Qinghai provinces). By comparison, we find that the YCDI is very applicable for monitoring drought intensity in the Yellow River Basin.

**Table 4:** Comparison of YCDI monitoring drought events and China Flood and Drought Bulletin events.

Drought monitoring by YCDI	Severe of drought monitored with YCDI	China flood and drought bulletin has a report of drought events	Severe of drought monitored with historical records
2006.02–2006.04	Severe drought	From March to April 2006, precipitation decreased, temperatures increased, and spring drought gradually developed in most areas north of the Yellow River.	Severe drought
2006.06–2007.02	Moderate drought	From June to November 2006, severe summer and autumn drought occurred in most regions of the country due to high temperatures and reduced precipitation.	Severe drought
		From January to February 2007, due to warm winter and low rainfall, drought developed rapidly in North China, Northwest China, and the Huanghuai region.	
2007.04–2007.09	Moderate drought	In March 2007, precipitation was scarce and the temperature increased rapidly. At the end of the month, the disaster area in the northern region was 9.3 × 106 ha.	Severe drought
		In May 2007, the national average temperature was 1.5 °C higher than in previous years, with severe drought in the north.	
		In June 2007, the national average temperature was 1.0 °C higher than in previous years.	
		From July to September 2007, the drought was alleviated with the increase of precipitation in the northern region.	
2007.11–2007.12	Moderate drought	From mid-October to late December 2007, precipitation in North China and parts of Northwest China decreased by 50%, resulting in severe winter drought.	Moderate drought
2008.03–2008.05	Moderate drought	In 2008, due to continued low precipitation and high temperatures, severe spring drought occurred in the northern region.	Moderate drought
2008.11–2009.04	Severe drought	In November 2008, precipitation in North China, Huanghuai, and East-Northwest China decreased by 5–9%. In December 2008, the drought in the Huang-Huai-Hai region continued to develop, with rain-free days reaching 80–120 in the most severe areas. In February 2009, precipitation occurred and drought alleviated. The disaster area of crops in the Yellow River Basin was about 1.02 × 107 hectares.	Severe drought
2009.09–2009.10	Mild drought	No drought	Near normal
2010.06–2010.08	Mild drought	In the west of Northeast China, most of North China, and the east of Northwest China, there were high temperatures and little rain, and the precipitation decreased by 3–8% compared with the same period in other years.	Mild drought
2010.10–2011.1	Mild drought	From October 2010 to February 2011, precipitation in winter wheat-producing areas decreased by 5–9% compared with the same period in other years. Reservoirs and ponds dried in Shaanxi, Gansu, and Henan Provinces.	Mild drought
2011.04–2011.08	Mild drought	No drought	Near normal
No drought	Near normal	In June 2012, the precipitation in the Huanghuai region decreased by 7–9% compared with the same period in other years and a severe drought occurred.	Mild drought
2013.02–2013.04	Moderate drought	In early 2013, the precipitation in eastern Shaanxi and Gansu decreased and drought developed rapidly. The affected area reached 1.2 × 10^6^ ha, and the drought was relieved in April.	Moderate drought
2013.11–2014.01	Moderate drought	From 2013 to April 14, the precipitation in winter wheat-production areas decreased by 4–6% compared with the same period in other years. The precipitation increased in April and the drought was relieved.	Moderate drought
2014.05–2014.08	Mild drought	From June to mid-August 2014, large-scale drought was caused by high temperatures and low rainfall north of the Yangtze River.	Mild drought
2014.11–2015.03	Moderate drought	From December 2014 to April 2015, precipitation in winter wheat-producing areas decreased by 40% in the same period in other years and by 6–8% locally. Precipitation increased in April and drought eased.	Moderate drought
2015.07–2015.08	Moderate drought	From June to August 2015, the precipitation decreased by 5–8% compared with the same period in other years, and temperature increased by 1–3 °C, resulting in short-term, high-intensity drought.	Moderate drought

**Note:**

Historical drought information from China Flood and Drought Bulletin and Meteorological Drought Yearbook.

**Table 5:** Correlation coefficients between the area of crop failure and drought intensity.

Area of crop failure						
Drought intensity	Gansu	Ningxia	Shaanxi	Shanxi	Henan	Inner Mongolia
	0.57	0.55	0.45	0.47	0.69	0.52

### Spatial-temporal analysis of drought in the Yellow River Basin

#### Drought trends

**Figure 3:**
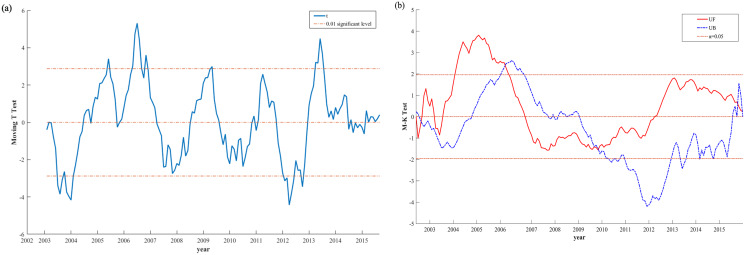
(A) M-K curves and (B) moving T test of the YCDI from 2002 to 2015.

The corresponding Moving T test (Fig. 3A) exceeds the significance level line in 2004, 2006, 2009 and 2013. As shown in Fig. 3B, the mutation results of M-K of YCDI had UF > 0, an increasing trend in 2002 to 2006 and 2012 to 2015, and the UF and UB curves had intersection points in 2003, 2009 and 2015. the mutation time points of YCDI based on M-K test were 2003, 2009, 2015. Taken together, YCDI underwent a mutation in 2009. The reason for the abrupt change is that the annual precipitation in northern China in 2009 was the lowest in nearly 30 years, and that in some areas was the lowest in nearly 50 years. From June 23 to 27, 2009, there was widespread high temperature weather in the north of China, and this high temperature weather affected a wide range and intensity, with local maximum temperatures reaching 40 °C to 43 °C in southern Hebei, northern Henan, Shandong, etc. (Jiang et al., 2012).

**Figure 4:**
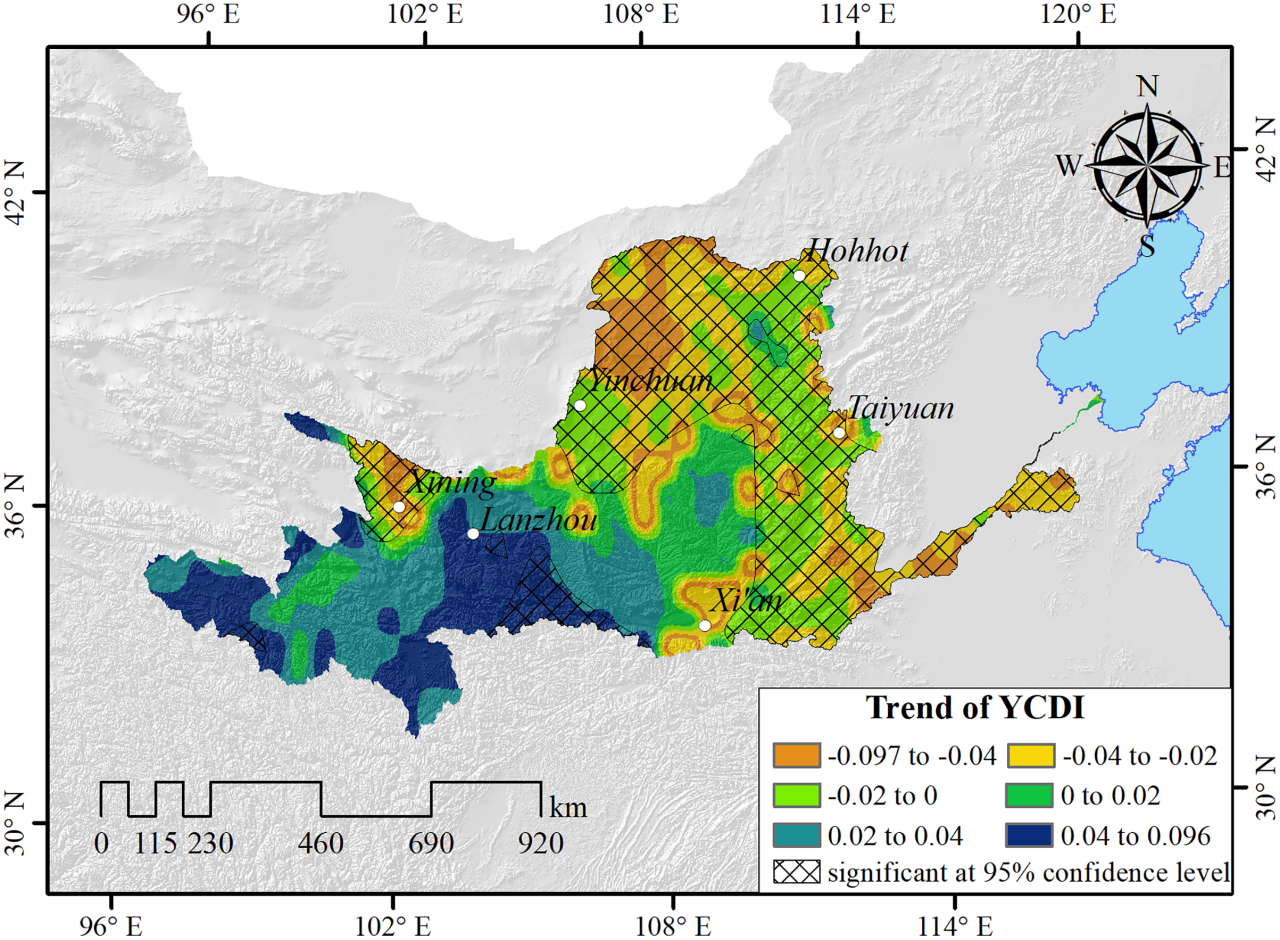
The spatial patterns of the YCDI. The crosshatch indicates that the trend is statistically significant at the 95% confidence level based on T-test.

The trends of drought occurrence in the Yellow River Basin also show obvious spatial patterns (Fig. 4). The YCDI of Henan, Shandong, and southwestern Shanxi in the lower reaches of the basin shows the most obvious decreasing trends, and the maximum decreasing rate reached 0.097/yr. The YCDI of central Inner Mongolia, southern Ningxia, central Shaanxi, eastern Gansu, northern Xining City in Qinghai Province, and the Yellow River coastal area of Shanxi Province showed a weak downward trend, indicating that the drought situation in the above areas is more serious. The YCDI in the southeast of Qinghai, the southwest of Gansu, and the north of Ningxia and Sichuan increased slightly, and the YCDI in the southeast of Qinghai increased most significantly with a maximum increase of 0.096/yr.

#### Drought frequency variation

**Figure 5:**
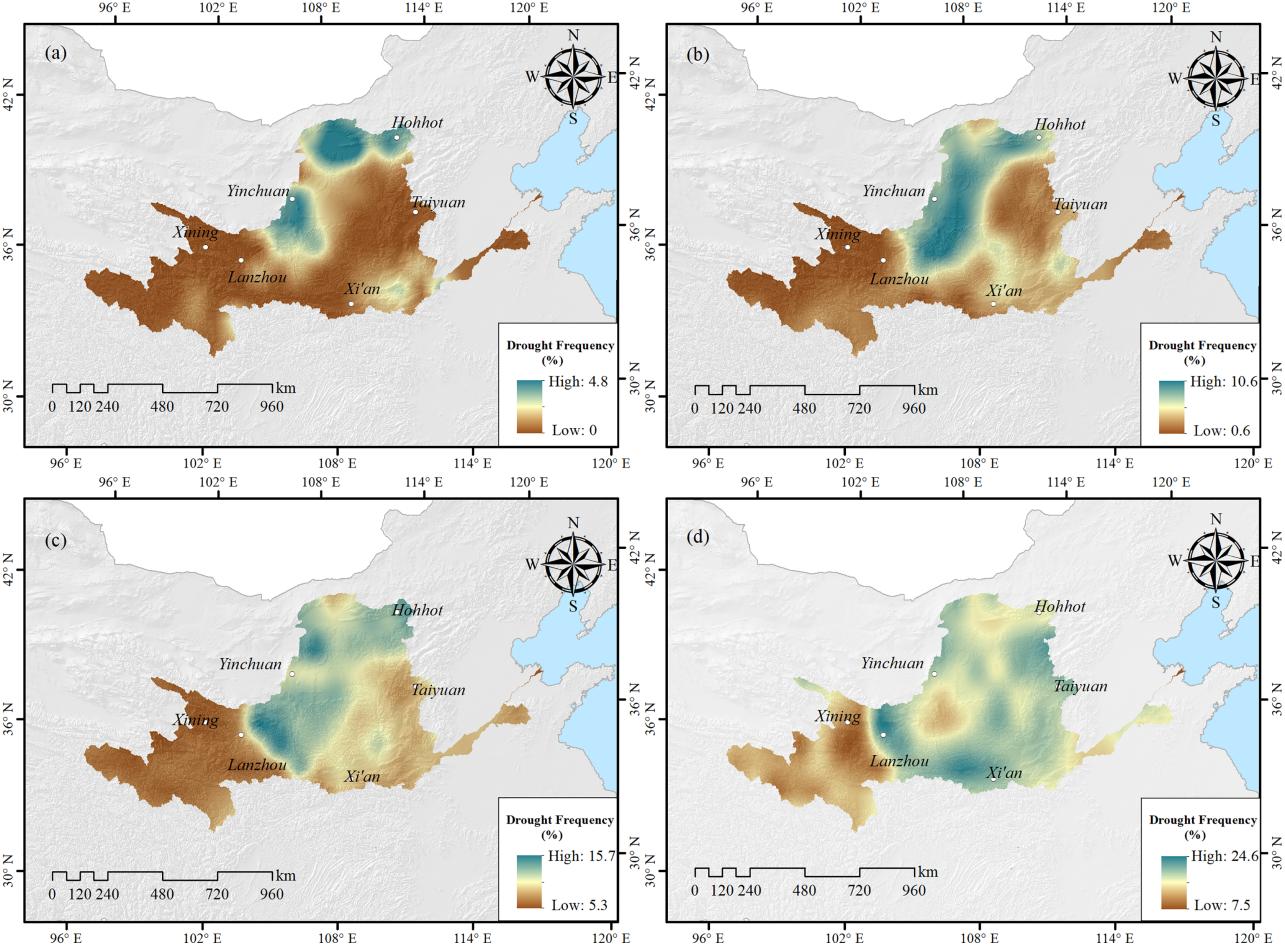
Spatial distribution of drought frequency of different grades in the Yellow River Basin. (A) Extreme drought; (B) severe drought; (C) moderate drought; (D) mild drought.

As shown in Fig. 5, the frequency of extreme drought in the Yellow River Basin was 0–4.83% (Fig. 5A), mainly distributed in northern Ningxia, central Inner Mongolia, the junction of Shanxi and Henan, and the Gansu and Sichuan areas. The frequency of severe drought was 0.65–10.64% (Fig. 5B), mainly distributed in Ningxia, southern Inner Mongolia, central Shaanxi, southwestern Shanxi, and parts of Henan and Gansu. The frequency of moderate drought was 5.31–15.75% (Fig. 5C), mainly distributed in Ningxia Hui Autonomous Region, eastern Gansu, and the Yellow River in Inner Mongolia. The frequency of mild drought was 7.26–24.58% (Fig. 5D), mainly distributed in central Shaanxi Province, eastern Gansu Province, northern Shanxi Province, northern Ningxia, and central Inner Mongolia. The frequency of different grades of drought in the Yellow River Basin during the research duration was: mild drought (15.84%), moderate drought (12.52%), severe drought (4.03%), and extreme drought (0.97%), respectively.

### Differences in response to typical drought occurrences between different drought indexes

**Figure 6:**
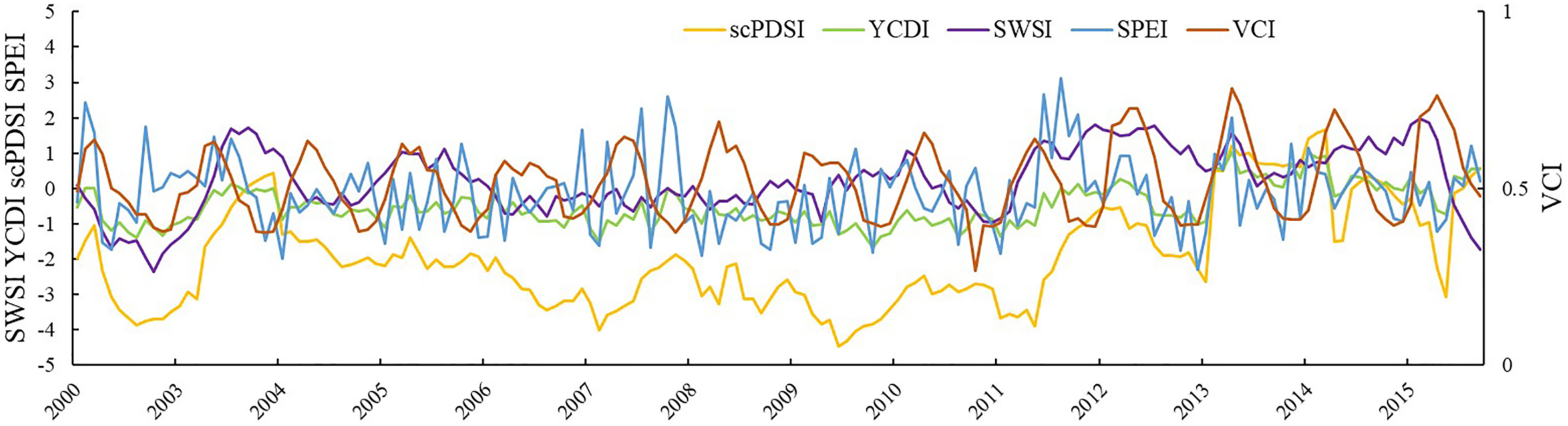
Comparison of YCDI, scPDSI, SPEI, SWSI, and VCI at Huining Station from 2002 to 2015.

From November 2008 to February 2009, severe droughts occurred in most parts of North China, eastern Northwest China, Huang-Huai, Jiang-Huai, Jianghan, and other northern winter wheat areas (Jiang et al., 2012). Compared with the same period in previous years, the precipitation decreased by 3–5%. The SPEI, scPDSI, VCI, SWSI, and YCDI drought index values corresponding to the Huining meteorological station were selected to explore the response differences of different drought indexes to drought events (Fig. 6). It can be seen that the trend of the YCDI is synchronous with SPEI and scPDSI, and it is highly sensitive to drought in the early stages. In the subsequent development, the SPEI index is weaker than YCDI and scPDSI in capturing persistent drought. The main reason is that the leading factor in the SPEI index is precipitation, and the evapotranspiration anomaly caused by temperature changes is a secondary factor. When precipitation occurs, the SPEI index will reduce the degree of drought evaluation and cannot achieve complete monitoring. The scPDSI index is slightly higher than other drought indexes because the scPDSI index not only includes precipitation and potential evapotranspiration but also considers the maximum effective water holding capacity of the soil. In arid areas or continuous drought events, the lower maximum effective water holding capacity will cause the scPDSI drought index to overestimate the drought grade.

The peak value of the SWSI lags after the YCDI, SPEI, and scPDSI. The reason is that the important supply source of land water reserves in the Yellow River Basin is precipitation, and the conversion of precipitation to water reserves has a lag, which is consistent with the time required for meteorological drought to develop into hydrological drought. Compared with other indexes, the SWSI index is not sensitive to severe drought, which may be limited by GRACE data quality accuracy. The VCI index shows strong annual variation characteristics and has a poor correlation with other indexes. It is not possible to fully describe drought characteristics solely based on vegetation growth state. Second, precipitation is not the most important factor in natural vegetation growth; instead, runoff and groundwater are more important. As a result, the VCI index will understate the severity of the drought. In conclusion, the YCDI developed in this paper combines the other four drought indexes, allowing for more accurate monitoring of drought occurrence, duration, and ending.

**Figure 7:**
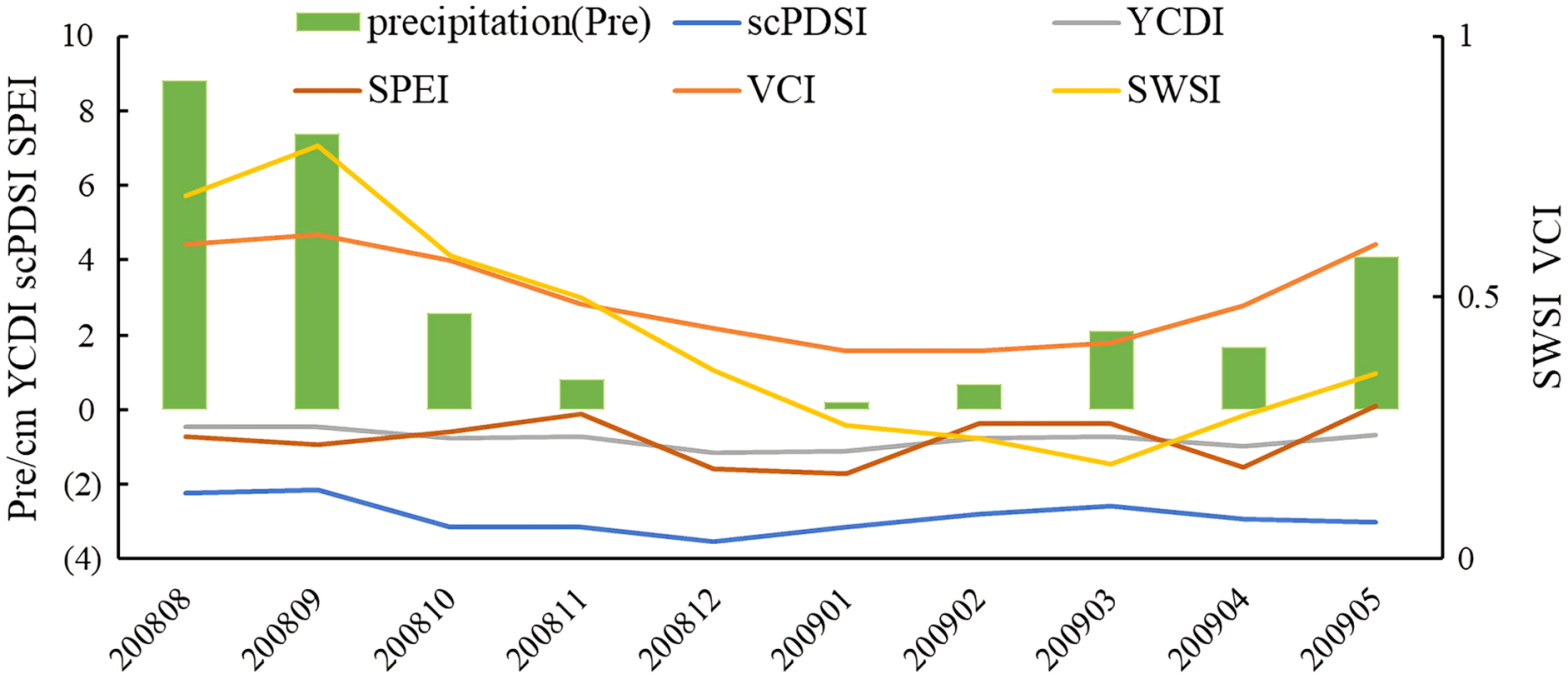
The trend of SPEI, scPDSI, SWSI, YCDI, VCI, and precipitation at Huining Station from August 2008 to May 2009.

By selecting YCDI, scPDSI, SPEI, SWSI, and VCI indexes and precipitation data from August 2008 to May 2009, the advantages of the YCDI in monitoring drought events were further verified (Fig. 7). From August 2008 to May of the following year, the precipitation at Huining station showed a strong temporal difference. From August, precipitation began to decrease. Except for VCI and SWSI, the other three indexes showed a one-month lag to the precipitation, and the drought signal was captured in September. The sensitivity of the SPEI index was the highest, followed by scPDSI, and YCDI was the weakest. In the subsequent development, YCDI was capable of monitoring drought, while the SPEI index was greatly affected by precipitation and maintained the same trend as precipitation. The scPDSI index maintained a downward trend from September to February, which overestimated the degree of drought. After January, each index increased, with scPDSI and SPEI indexes having the most significant increases. The VCI index began to increase in March, and the SWSI index had the largest increase in March. Because precipitation needs a certain period to recharge runoff and groundwater, the monitoring results have a certain delay. The comparison shows that the YCDI has a better continuous monitoring ability for drought events.

**Figure 8:**
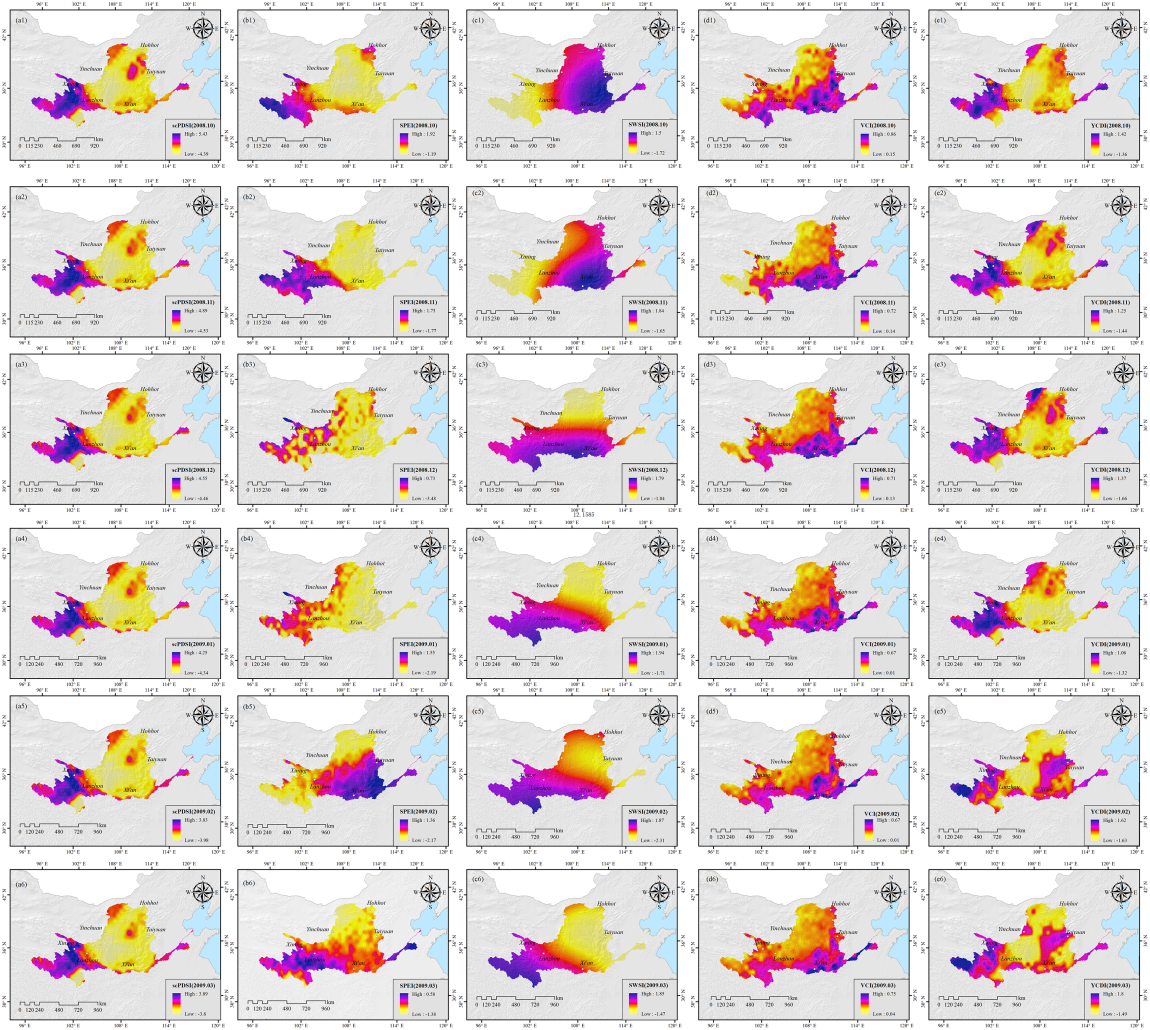
A comparison of five drought indicators for drought monitoring (A1-D6).

The spatial change of drought in the Yellow River Basin as monitored by various drought indexes from October 2008 to March 2009 is shown in Fig. 8. The differences can be attributed to both the index compositions and climate change. The scPDSI is the most analogous to the YCDI, however, when compared to other indexes that monitored drought development, changes are not as clear. The key reason for this is that scPDSI generates parameters using historical data from each station and more objectively examines the regional variability of parameters (Shrestha et al., 2020). The SWSI constructed based on TWSA lags slightly behind other drought indexes for about two months. The monitoring results of SWSI in the eastern portion of the basin from December 2008 to March the following year were not consistent with those of other drought indexes. Soil moisture in North China is influenced by both natural and human influences. The exploitation of groundwater provides for 70% of total water resource use in the North China plain and irrigation water accounts for 81.7% of agricultural water. In the North China plain, 79.7% of groundwater is used directly for agricultural irrigation (Yang et al., 2017). Irrigation will invariably have an impact on shallow soil water and vegetation growth. As a result, VCI, scPDSI, and SPEI are unable to adequately depict drought in North China.

Limited by the number of meteorological stations, YCDI also failed to effectively monitor drought in North China. Related studies have shown that the applicability of SPEI in humid areas is high, but it is relatively poor in arid and semi-arid areas because SPEI will overestimate the contribution of temperature anomalies to drought (Liu, Kang & Wang, 2016). VCI reflects drought from the growth of vegetation, but other factors, such as pests and diseases, floods, improper fertilization, and lack of irrigation, will also lead to poor vegetation growth (Jibril et al., 2022). As a consequence, VCI only represents comprehensive information on vegetation growth and cannot specifically reflect drought information, particularly in agricultural producing areas, as is shown in Fig. 8. The comparison reveals that the YCDI incorporates the benefits of each indicator and that drought monitoring results are consistent with official reports.

## Discussion

### Factors influencing drought in the Yellow River Basin

The trend and frequency of drought in the Yellow River Basin have significant regional variation. From the spatial distribution and frequency of YCDI, we can see that the eastern and central parts of the Yellow River basin belong to drought-prone areas (Figs. 3 and 4). This conclusion is consistent with the findings of Huang et al. (2020) and Peng et al. (2011). In addition, we found that the frequency of droughts in the Yellow River basin gradually increases from south to north, which is also verified with the results of She, Xia & Du (2012) and Xu, Yang & Yang (2014). Therefore, the YCDI proposed in this study is scientifically reliable and has strong applicability compared with the results of existing studies on drought in the Yellow River basin.

Under the combined effect of climate warming and human activities, the whole Yellow River basin is moving toward a warming and drying trend, and extreme climate events are becoming more frequent. As a result, this has led to an increased risk of drought in the Yellow River basin, especially in the middle and lower reaches of the basin. Over the past 70 years, residential water use in the Yellow River basin has jumped from 20% to 80% of runoff, with surface water resource utilization well above the 40% runoff health alert (Piao et al., 2010). However, due to the large east-west span of the Yellow River basin and significant climatic differentiation, the causes of disasters also differ significantly. The drought in the lower Yellow River basin is mainly influenced by human activities. This region is one of the most important food resources in China, and crop irrigation leads to a continuous decrease in effective soil water holding capacity, which corresponds to the trend of YCDI. In contrast, the high-frequency drought in the Ningxia-Inner Mongolia region in the upper part of the basin is caused by both human activities and climate change. The Ningxia-Inner Mongolia section of the upper Yellow River has a temperate continental climate with low precipitation (200–300 mm/year) and high evaporation, where grassland is the primary vegetation type (Wu et al., 2018). Due to the considerable variations in regulating climate, soil, and water conservation between single vegetation ecosystems and complex community ecosystems, as well as the loss of limited precipitation in the form of runoff, effective water control is not achievable. Secondly, energy bases with high water consumption, such as Ningxia Eastern Energy Base and Hohhot-Baotou-Erdos Economic Circle. Regional droughts are becoming more frequent and their intensity is growing as the regional economy continues to expand and urbanization accelerates (Ma et al., 2019).

### The limitations of YCDI for drought monitoring

To construct a comprehensive drought index, it is necessary to integrate meteorological, hydrological, and vegetation factors at different temporal and spatial scales. Different drought indexes have varied monitoring results for drought events, which depend on the calculation method and underlying data (Feng et al., 2019). The four single indexes used in this paper describe drought from aspects of meteorology, vegetation, and hydrology, but the ability of the VCI index to monitor drought is limited in the winter dormancy period of vegetation. The resolutions of the four single indexes were inconsistent. After the unified resolution, the YCDI resolution is 1° × 1° which can be used for effective monitoring of drought on a large scale but is still not sensitive to drought on small scales.

Because of global climate change and land-water cycle disruptions, extreme hydrological events are more common (Levizzani & Cattani, 2019). Flash droughts are very short (*e.g*., a few days or weeks) and are high magnitude events, and are often accompanied by a heatwave, soil moisture loss, and evapotranspiration increase, thus having a serious impact on crop yield and water supply (Wang & Yuan, 2018; Zhang et al., 2022). The YCDI on a monthly scale has poor adaptability to unexpected drought, and it cannot match the eight-day or even daily scale drought monitoring requirements. In the future, our focus will be on developing a set of short-term drought monitoring indexes.

Compared with other research results, the main contribution of this paper is the introduction of the SWSI. Its advantage is that drought can be considered comprehensively from the water stored in each layer of land and it considers the impact of human activities on hydrological flux. In the Loess area of the middle reaches of the Yellow River Basin, the complex correlation between surface water and groundwater makes it difficult for most hydrological models and observation methods to capture the accurate water change in the region. TWSA is an important indicator of regional water resources changes, reflecting the changes in surface water, groundwater and river and lake water (Tapley et al., 2019), the SWSI has the advantages of uniform distribution and scale. In general, SWSI has great application potential and can reflect the temporal and spatial variation of drought in time and space, especially for large-scale areas with hydrological and meteorological data (Han et al., 2021b). However, the limitation of the SWSI is that the accuracy and stability of its monitoring results are affected by the quality of GRACE data. The TWSA time series is relatively short, resulting in its multi-year average not being representative. Because of the periodic variations of TWSA, some scholars believe that the time series of TWSA over 30 years is preferable (Deng et al., 2020). These factors make the use of SWSI for drought monitoring and analysis still uncertain, but the GRACE gravity satellite data remains the most effective technical means for monitoring land water reserves (Nie et al., 2018). With the operation of GRACE-Follow on, the future construction of a comprehensive drought index based on a long-term series of the SWSI will improve the monitoring accuracy.

## Conclusions

Drought indexes that rely on a single variable are unable to effectively reflect drought conditions. As a result, developing an integrated indicator for drought evaluation is critical. In this study, a comprehensive drought monitoring index YCDI, with SWSI, scPDI, VCI, and SPEI as raw data, were constructed. Compared with typical drought events, the applicability of the YCDI was verified, and on this basis, the temporal and spatial distribution of drought in the basin were analyzed. The main conclusions are:

1) YCDI has stronger ability to monitor drought process. In terms of time scale and drought degree, the monitoring results based on YCDI were similar with data presented in the China Flood and Drought Bulletin and Meteorological Drought Yearbook, reaching ~87% and ~69%, respectively. The correlation between drought intensity and crop harvest area was 0.56.
2) By the combined analysis of the Mann-Kendall test and moving T test, it was found that the abrupt change of YCDI index at the time of 2009.
3) The YCDI of the basin showed a weak downward trend in the past 14 years, that is, the drought risk has increased. The drought-prone areas are mainly distributed in the upper reaches of the basin, such as Gansu, Ningxia, and Inner Mongolia; the Guanzhong Plain in the middle reaches; and in the lower reaches, Henan and Shandong. The area west of the Lanzhou section maintained a state of no or light drought.
4) The YCDI of Henan and Shandong in the lower reaches of the basin decreased at a maximum rate of 0.097/yr, and the index of Qinghai in the upper reaches increased at a maximum rate of 0.096/yr.
5) In the Yellow River Basin, the frequency of light drought, moderate drought, severe drought, and extreme drought was 15.84%, 12.52%, 4.03%, and 0.97%, respectively. The frequency of drought in Ningxia, Inner Mongolia, and central Shaanxi was the highest.

## Supplemental Information

10.7717/peerj.13560/supp-1 Supplemental Information 1 GRACE data This article uses Gravity Recovery and Climate Experiment (GRACE) Level-2 gravity field model data provided by the Center for Space Research (CSR) of the University of Texas (https://www.csr.utexas.edu). The effects of solid tides, sea tides, polar tides, non-tidal atmosphere and ocean mass generated by the Earth’s rotation, and the gravity disturbance caused by extraterrestrial stars were removed from the data. The GFC files can be opened using ArcGIS 10.4, and the software is authorized by ESRI (https://www.geoscene.cn/).

10.7717/peerj.13560/supp-2 Supplemental Information 2 MODIS NDVI data The MODIS NDVI data (MOD13A3) with 1 km spatial resolution was downloaded from https://ladsweb.modaps.eosdis.nasa.gov for 2002 to 2015. The ADF files can be opened using ArcGIS 10.4, and the software is authorized by ESRI (https://www.geoscene.cn/).

10.7717/peerj.13560/supp-3 Supplemental Information 3 scPDSI dataset The scPDSI dataset (self-calibrating Palmer drought severity index based on Penman-Monteith) came from the Climate Research Unit (CRU) of the University of East Anglia (http://www.cru.uea.ac.uk/data); the spatial resolution is 0.5° × 0.5° and the period from 1901 to 2017. The ADF files can be opened using ArcGIS 10.4, and the software is authorized by ESRI (https://www.geoscene.cn/).

## Additional Information and Declarations

### Data Availability

The following information was supplied regarding data availability: The monthly total precipitation data and monthly average temperature data for 2002–2015 are available at the Meteorological Data Sharing Service System of China Meteorological Administration (http://data.cma.cn/).

## References

[ref-1] Andrew W, Jodie M, Annika K, Sven K (2022). Using soil-moisture drought indices to evaluate key indicators of agricultural drought in semi-arid Mediterranean Southern Africa. Science of the Total Environment.

[ref-2] Aoa B, Ma Z, Zza B, Fsb C (2020). Natural and anthropogenic influences on the recent droughts in Yellow River Basin China. Science of the Total Environment.

[ref-3] Araghinejad S (2011). An approach for probabilistic hydrological drought forecasting. Water Resources Management.

[ref-4] Ayantobo OO, Li Y, Song S (2019). Multivariate drought frequency analysis using four-variate symmetric and asymmetric archimedean copula functions. Water Resources Management.

[ref-5] Beguería S, Vicente-Serrano SM, Reig F, Latorre B (2014). Standardized precipitation evapotranspiration index (SPEI) revisited: parameter fitting, evapotranspiration models, tools, datasets and drought monitoring. International Journal of Climatology.

[ref-6] Dai M, Huang S, Huang Q, Leng G, Guo Y, Wang L, Fang W, Li P, Zheng X (2020). Assessing agricultural drought risk and its dynamic evolution characteristics. Agricultural Water Management.

[ref-7] Deng Z, Wu X, Wang Z, Li J, Chen X (2020). Drought monitoring based on GRACE data in the Pearl River Basin, China[J]. Transactions of the Chinese Society of Agricultural Engineering.

[ref-8] Erfurt M, Skiadaresis G, Tijdeman E, Blauhut V, Stahl K (2020). A multidisciplinary drought catalogue for southwestern Germany dating back to 1801. Natural Hazards and Earth System Sciences.

[ref-9] Felicia C, Omid M, Amir A (2021). Evidence of anthropogenic impacts on global drought frequency, duration, and intensity. Nature Communications.

[ref-10] Feng P, Wang B, Liu D, Yu Q (2019). Machine learning-based integration of remotely-sensed drought factors can improve the estimation of agricultural drought in South-Eastern Australia. Agricultural Systems.

[ref-11] Han H, Bai J, Yan J, Yang H, Ma G (2021a). A combined drought monitoring index based on multi-sensor remote sensing data and machine learning. Geocarto International.

[ref-12] Han Z, Huang S, Huang Q, Leng G (2021b). GRACE-based high-resolution propagation threshold from meteorological to groundwater drought. Agricultural and Forest Meteorology.

[ref-13] Han Z, Huang S, Huang Q, Leng G, Wang H, He L, Fang W, Li P (2019). Assessing GRACE-based terrestrial water storage anomalies dynamics at multi-timescales and their correlations with teleconnection factors in Yunnan Province China. Journal of Hydrology.

[ref-14] He J, Yang X, Li J, Jin J, Wei Y, Chen X (2015). Spatiotemporal variation of meteorological droughts based on the daily comprehensive drought index in the Haihe River basin China. Natural Hazards.

[ref-15] Huang S, Chang J, Leng G, Huang Q (2015). Integrated index for drought assessment based on variable fuzzy set theory: a case study in the Yellow River basin China. Journal of Hydrology.

[ref-16] Huang L, Wang J, Chen X (2022). Ecological infrastructure planning of large river basin to promote nature conservation and ecosystem functions. Journal of Environmental Management.

[ref-17] Huang S, Wang L, Wang H, Huang Q, Leng G, Fang W, Zhang Y (2019). Spatio-temporal characteristics of drought structure across China using an integrated drought index. Agricultural Water Management.

[ref-18] Huang S, Zheng X, Ma L, Wang H, Guo Y (2020). Quantitative contribution of climate change and human activities to vegetation cover variations based on GA-SVM model. Journal of Hydrology.

[ref-19] Hurtado SI, Zaninelli PG, Agosta EA, Ricetti L (2021). Infilling methods for monthly precipitation records with poor station network density in Subtropical Argentina. Atmospheric Research.

[ref-20] Jeng H, Englande A, Bakeer R, Bradford H (2005). Impact of urban stormwater runoff on estuarine environmental quality. Estuarine, Coastal and Shelf Science.

[ref-21] Jiang D, Fu J, Zhuang D, Xu X (2012). Dynamic drought-remote sensing monitoring in north China from 2008 to 2009. Journal of Natural Disasters.

[ref-22] Jibril AM, Nurfarhana R, Zed Z, Khairudin N, Mohamed RB, Melissa MF, Ain KN, Fredolin T (2022). Index-based insurance and hydroclimatic risk management in agriculture: a systematic review of index selection and yield-index modelling methods. International Journal of Disaster Risk Reduction.

[ref-23] Joses H, Tayfun T, Sameer A, Hyungwon C, Adam C-C (2019). Moving beyond P values: data analysis with estimation graphics. Nature Methods.

[ref-24] Jyolsna PJ, Kambhammettu B, Gorugantula S (2021). Application of random forest and multi linear regression methods in downscaling GRACE derived groundwater storage changes. Hydrological Sciences Journal/Journal des Sciences Hydrologiques.

[ref-72] Kogan FN (1995). Application of vegetation index and brightness temperature for drought detection[J]. Advances in Space Research.

[ref-25] Leng G, Tang Q, Rayburg S (2015). Climate change impacts on meteorological, agricultural and hydrological droughts in China. Global & Planetary Change.

[ref-26] Levizzani V, Cattani E (2019). Satellite remote sensing of precipitation and the terrestrial water cycle in a changing climate. Remote Sensing.

[ref-27] Liu S, Kang W, Wang T (2016). Drought variability in Inner Mongolia of northern China during 1960–2013 based on standardized precipitation evapotranspiration index. Environmental Earth Sciences.

[ref-28] Liu X, Zhu X, Zhang Q, Yang T, Sun P (2020). A remote sensing and artificial neural network-based integrated agricultural drought index: index development and applications. Catena.

[ref-29] Loon V, Anne F (2015). Hydrological drought explained. Wiley Interdisciplinary Reviews: Water.

[ref-30] Lu J, Yu K, Li Z, Li P, Xu G, Cheng Y, Zhang X, Yang Z (2021). The effect of meteorological drought on vegetation cover in the Yellow River Basin, China. International Journal of Climatology.

[ref-31] Ma S, Zhang B, Yang M, Wang G, Cao B (2019). Analysis on drought and flood disasters in North China Plain from 1901 to 2015 based on EEMD. Journal of Arid Land Resources and Environment.

[ref-32] Mishra AK, Singh VP (2010). A review of drought concepts. Journal of Hydrology.

[ref-33] Moyé L, Kapadia AS (1995). Predictions of drought length extreme order statistics using run theory. Journal of Hydrology.

[ref-34] Ni J, Li P, Wei C, Xie D (2009). Potentialities evaluation of regional land consolidation based on AHP and entropy weight method. Transactions of the Chinese Society of Agricultural Engineering.

[ref-35] Nie N, Zhang W, Chen H, Guo H (2018). A global hydrological drought index dataset based on gravity recovery and climate experiment (GRACE) data. Water Resources Management.

[ref-37] Palmer WC (1965). Meteorological Drought.

[ref-38] Peng G, Xia J, Ma X, Ma J (2011). Analysis on drought frequency distribution and digital characteristics of number of turns of the Yellow River basin. Yellow River.

[ref-39] Piao S, Ciais P, Huang Y, Shen Z, Peng S, Li J, Zhou L, Liu H, Ma Y, Ding Y, Friedlingstein P, Liu C, Tan K, Yu Y, Zhang T, Fang J (2010). The impacts of climate change on water resources and agriculture in China. Nature.

[ref-40] Pulwarty RS, Sivakumar M (2014). Information systems in a changing climate: early warnings and drought risk management. Weather & Climate Extremes.

[ref-41] Quiring SM, Ganesh S (2010). Evaluating the utility of the Vegetation Condition Index (VCI) for monitoring meteorological drought in Texas. Agricultural and Forest Meteorology.

[ref-36] Rahmati O, Falah F, Dayal KS, Deo RC, Mohammadi F, Biggs T, Moghaddam DD, Naghibi SA, Bui DT (2020). Machine learning approaches for spatial modeling of agricultural droughts in the south-east region of Queensland Australia. Science of the Total Environment.

[ref-42] Scanlon BR, Zhang Z, Save H, Wiese DN, Landerer FW, Long D, Longuevergne L, Chen J (2016). Global evaluation of new GRACE mascon products for hydrologic applications. Water Resources Research.

[ref-43] Shakeel A, Muhammad K, Ruixia D, Xiangping M, Haiqi W, Irshad A, Shah F, Qingfang H (2019). Exogenous melatonin confers drought stress by promoting plant growth, photosynthetic capacity and antioxidant defense system of maize seedlings. PeerJ.

[ref-44] She DX, Xia J, Du H (2012). Spatio-temporal analysis and multi-variable statistical models of extreme drought events in Yellow River Basin, China. Journal of Basic Science and Engineering.

[ref-45] Shrestha A, Rahaman M, Kalra A, Thakur B, Maheshwari P (2020). Regional climatological drought: an assessment using high-resolution data. Hydrology.

[ref-46] Tapley BD, Watkins MM, Flechtner F, Reigber C, Bettadpur S, Rodell M, Sasgen I, Famiglietti JS, Landerer FW, Chambers DP (2019). Contributions of GRACE to understanding climate change. Nature Climate Change.

[ref-47] Tayyebeh M, Maryam M, Mohseni SM, Soleimani SF, Miglietta MM (2020). Meteorological drought analysis using copula theory and drought indicators under climate change scenarios (RCP). Meteorological Applications.

[ref-48] Trenberth KE, Dai A, Gerard V, Jones PD, Barichivich J, Briffa KR, Sheffield J (2013). Global warming and changes in drought. Nature Climate Change.

[ref-49] Vicente-Serrano SM, Beguería S, López-Moreno JI (2010). A multiscalar drought index sensitive to global warming: the standardized precipitation evapotranspiration index. Journal of Climate.

[ref-50] Wang H, Huang S, Di D, Wang Y, Zhang F (2021). Study on the spatial distribution of water resource value in the agricultural system of the Yellow River Basin. Water Policy.

[ref-51] Wang F, Wang Z, Yang H, Di D, Zhao Y, Liang Q, Hussain Z (2020). Comprehensive evaluation of hydrological drought and its relationships with meteorological drought in the Yellow River basin China. Journal of Hydrology.

[ref-52] Wang L, Ying G-G, Chen F, Zhang L-J, Zhao J-L, Lai H-J, Chen Z-F, Tao R (2012). Monitoring of selected estrogenic compounds and estrogenic activity in surface water and sediment of the Yellow River in China using combined chemical and biological tools. Environmental Pollution.

[ref-53] Wang L, Yuan X (2018). Two types of flash drought and their connections with seasonal drought. Advances in Atmospheric Sciences.

[ref-54] Wu Y, Dong S, Huang H, Zhai J, Li Y, Huang D (2018). Quantifying urban land expansion dynamics through improved land management institution model: application in ningxia-inner Mongolia China. Land Use Policy.

[ref-55] Wu H, Hayes M, Weiss A, Hu Q (2001). An evaluation of the standardized precipitation index, the China-Z index and the statistical Z-Score. International Journal of Climatology: A Journal of the Royal Meteorological Society.

[ref-56] Xiao M, Zhang Q, Singh VP, Liu L (2016). Transitional properties of droughts and related impacts of climate index in the Pearl River basin China. Journal of Hydrology.

[ref-57] Xie F, Gu J, Lin Z (2014). Assessment of aquatic ecosystem health based on principal component analysis with entropy weight: a case study of wanning reservoir (Hainan Island China). Chinese Journal of Applied Ecology.

[ref-58] Xu K, Yang D, Yang H (2014). Spatio-temporal variation of drought in China during 1961-2012: a climatic perspective. Journal of Hydrology.

[ref-59] Yang Y, Long D, Guan H, Scanlon BR, Simmons CT, Jiang L, Xu X (2015b). GRACE satellite observed hydrological controls on interannual and seasonal variability in surface greenness over mainland Australia. Journal of Geophysical Research Biogeosciences.

[ref-60] Yang Q, Mingxing LI, Zheng ZY, Zhuguo MA (2017). Regional applicability of seven meteorological drought indices in China. Science China Earth Sciences.

[ref-61] Yang T, Zhou X, Yu Z, Krysanova V, Wang B (2015a). Drought projection based on a hybrid drought index using artificial neural networks. Hydrological Processes.

[ref-62] Yimam KA, Shao G, Wang X, Wu S (2021). Quantification of drought severity change in Ethiopia during 1952–2017. Environment, Development and Sustainability.

[ref-63] Yu Y, Wang J, Cheng F, Deng H, Chen S (2020). Drought monitoring in Yunnan Province based on a TRMM precipitation product. Natural Hazards.

[ref-64] Yue S, Wang C (2004). The Mann-Kendall test modified by effective sample size to detect trend in serially correlated hydrological series. Water Resources Management.

[ref-65] Yue Q, Yang D, Lei H, Kai X, Xu X (2015). Comparative analysis of drought based on precipitation and soil moisture indices in Haihe basin of North China during the period of 1960–2010. Journal of Hydrology.

[ref-66] Zhang X, Chen N, Li J, Chen Z, Niyogi D (2017). Multi-sensor integrated framework and index for agricultural drought monitoring. Remote Sensing of Environment.

[ref-67] Zhang X, Duan Y, Duan J, Jian D, Ma Z (2022). A daily drought index based on evapotranspiration and its application in regional drought analyses. Science China (Earth Sciences).

[ref-68] Zhang L, Liu Y, Ren L, Jiang S, Yang X, Yuan F, Wang M, Wei L (2019). Drought monitoring and evaluation by ESA CCI soil moisture products over the Yellow River Basin. IEEE Journal of Selected Topics in Applied Earth Observations and Remote Sensing.

[ref-69] Zhao M, Huang S, Huang Q, Wang H, Leng G, Xie Y (2019). Assessing socio-economic drought evolution characteristics and their possible meteorological driving force. Geomatics, Natural Hazards and Risk.

[ref-70] Zhou K, Li J, Zhang T, Kang A (2021). The use of combined soil moisture data to characterize agricultural drought conditions and the relationship among different drought types in China. Agricultural Water Management.

[ref-71] Zhu Y, Liu Y, Ma X, Ren L, Singh V (2018). Drought analysis in the Yellow River basin based on a short-scalar palmer drought severity index. Water.

